# Coupling of Defect Modes in Cholesteric Liquid Crystals Separated by Isotropic Polymeric Layers

**DOI:** 10.3390/polym10070805

**Published:** 2018-07-23

**Authors:** Shaohua Gao, Yanzi Zhai, Xinzheng Zhang, Xiao Song, Jiayi Wang, Irena Drevensek-Olenik, Romano A. Rupp, Jingjun Xu

**Affiliations:** 1 The MOE Key Laboratory of Weak-Light Nonlinear Photonics, TEDA Institute of Applied Physics and School of Physics, Nankai University, Tianjin 300457, China; gaosh@mail.nankai.edu.cn (S.G.); yanzizhai@mail.nankai.edu.cn (Y.Z.); 2120170195@mail.nankai.edu.cn (X.S.); 2120170196@mail.nankai.edu.cn (J.W.); romano.rupp@univie.ac.at (R.A.R.); jjxu@nankai.edu.cn (J.X.); 2 Faculty of Mathematics and Physics, University of Ljubljana, Jadranska 19 and Department of Complex Matter, J. Stefan Institute, Jamova cesta 39, 1000 Ljubljana, Slovenia; 3 Faculty of Physics, Vienna University, Boltzmanngasse 5, A-1090 Wien, Austria; 4 Synergetic Innovation Center of Chemical Science and Engineering, Tianjin 300071, China

**Keywords:** cholesteric liquid crystals, optical defect modes, mode coupling, photonic density of state

## Abstract

Cholesteric liquid crystal structures with multiple isotropic defect layers exhibit localized optical modes (defect modes). Coupling effects between these modes were simulated using the finite difference time domain method. Analogous to the well-known result of the tight-binding approximation in solid state physics, splitting of the defect modes takes place, as soon as the structure contains more than one defect layer. The dispersion relation of the mini-bands forming within the photonic band gap of the structure is calculated numerically. The structures might have promising applications for multiwavelength filters and low-threshold lasers.

## 1. Introduction

Cholesteric liquid crystals (CLCs) have a periodic helical structure and thus can be regarded as one-dimensional (1D) photonic crystals but with a photonic bandgap (PBG) only for circularly polarized light of the same handedness as the helix. Since the emission rate of an optical eigenmode is, according to Fermi’s golden rule, proportional to its density of states (DOS) [1] and since the photonic DOS is particularly high at the edge of a PBG, lasing can occur if laser dyes are doped into a CLC [2]. This is one of the reasons why optical properties of CLCs have been widely investigated in recent years. Because of their strong sensitivity to temperature, electric field, and mechanical stress, CLCs have found applications in LCDs [3], sensors [4], and tunable lasers [5,6,7].

Yang et al. [8] introduced an isotropic layer that disturbs the periodic structure of a CLC. Simulations showed that such a defect layer can exhibit a corresponding optical defect mode within the PBG. Kopp and Genack contributed with a study of a twist defect [9] and Schmidtke and Stille with a discussion of different defect types in CLCs [10]. Because of the high Q-factor of defect modes, research soon focused on the attractive perspective of low threshold lasing. Using polymeric CLCs, Schmidtke et al. were the first to experimentally demonstrate twist defect mode lasing [11] and later reported also on an anisotropic defect layer in a CLC medium [12]. Although lasing in CLCs with an isotropic defect layer was the first model case to be studied theoretically, it was the last to be realised experimentally because of the difficulty to control the thickness of the isotropic layer [13].

Ha et al. theoretically introduced Fibonacci phase defects in a CLC structure, thus constructing a quasi-periodic system forming multiple PBGs [14]. Subsequently, they realized a quasi-periodic system using single-pitch CLCs and isotropic films of polyvinyl alcohol and fabricated a red-green-blue reflector [15]. There were further theoretical studies that considered reflectors [16,17,18], studied optical properties of composites of CLCs and isotropic layers [19,20,21,22] or calculated the DOS of a stack structure of CLCs and isotropic medium [23,24]. However, up until now, very little attention has been paid to the coupling of defect modes in such composite assemblies.

Defect cavities can be seen as the optical analogues of atoms in solid state physics. As long as they are well separated, they behave like isolated atoms. Similarly, well-separated optical resonators within a waveguide structure exhibit localized optical modes. As they approach each other, atomic wave functions, and similarly the optical fields of the modes, overlap with each other and coupling occurs. In the optical example, photons then can propagate because of the existence of the coupling effects. Yariv et al. e.g., applied the tight-binding (TB) approximation to investigate the coupling of resonators in a waveguide [25].

As the analogy suggests, the coupling of defect modes in 3D photonic crystals also can be adequately tackled by the TB theory known from solid state physics [26], and there is indeed a host of works that make use of it [27,28,29,30]. Studies of the coupling between defect modes were so far focused mainly on systems consisting of isotropic materials.

In this paper, we investigate the coupling of defect modes in a system of multiple defect layers (MDL) in a CLC (MDL-CLC) theoretically. The eigenmodes of this system have circular polarization. Optical transmission of the MDL-CLC structures is numerically calculated with the finite difference time domain (FDTD) method. By analysing the behaviour of the defect modes via analogy with the TB theory, we discuss mode coupling effects in different MDL-CLC structures. We propose using MDL-CLC structures for low threshold lasing. Single mode and multi-mode lasing in the visible light range of the spectrum may be realized by changing the number of defect layers in the MDL-CLC assembly.

## 2. The MDL-CLC Model

**Figure 1:**
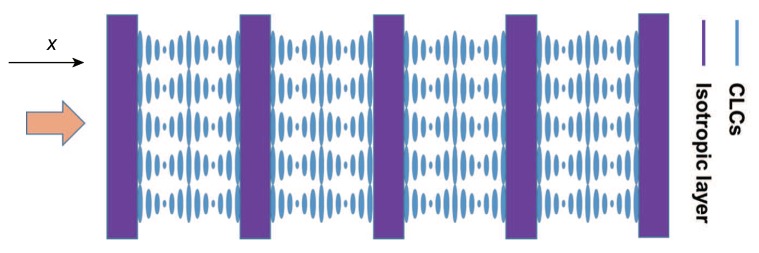
Sketch of the MDL-CLC structure.

The MDL-CLC structure is a 1D periodic composite structure formed by subsequent CLC layers of thickness $d_{C}$ and isotropic layers of thickness $d_{I}$. The repeat period of one unit of the MDL-CLC composite structure is $d=d_{C}+d_{I}$. We assume *D* is the thickness of the whole composite structure. The isotropic layers (purple) divide the CLC medium (blue) into several separate “cells”, as shown in Figure 1. All CLC helices are anchored at the walls of the “cells”, i.e., at the isotropic layers with the same chiral twist angle zero. Thus, the thickness $d_{C}$ of each CLC cell is an integer multiple of the half pitch *p/2*. In the calculations below, the thickness $d_{I}$ of the isotropic layer as well as the number of repeat units are free parameters that are varied to study the effect on the coupling of the defect modes. To be realistic, material parameters were chosen for the calculations such that they correspond for the CLC to the mixture of 60 wt. % E7 (nematic liquid crystal, Shijiazhuang Chengzhi Yonghua Display Material Co., Shijiazhuang, China) with 40 wt. % CB15 (chiral agent, Shijiazhuang Chengzhi Yonghua Display Material Co.) and for the isotropic medium to the photoresist SU-8 (MicroChem Corp., Westborough, MA, USA). The ordinary and extraordinary refractive indices of the CLC medium were chosen to be $n_{o}$ = 1.576 and $n_{e}$ = 1.691 and the pitch to be *p* = 348 nm. A refractive index of 1.7 was selected for the isotropic polymeric layers. The PBG ranges from $n_{o}p$ to $n_{e}p$, but occurs only for right circularly polarized light because CB15 is a right handed chiral agent.

One might wonder about the feasibility of the ideal MDL-CLC model structure defined above. It assumes perfect alignment of all CLC layers. This is the requirement for applications where high optical quality is requested. In our previous work, we invented a method to solve this technological key problem: SU-8 polymer ribbons were fabricated by a two-photon polymerization-based direct laser writing technique that by standing-wave interference generates surface relief gratings on both sidewalls of each ribbon [31,32]. If a CLC material is filled into such a polymeric scaffold, the nematic liquid crystal component (e.g., E7) efficiently aligns such that its LC director is perfectly oriented along the groove structure of the surface relief gratings. Hence, the helical axis of a CLC is aligned precisely perpendicular to the polymer ribbons, as in the model shown in Figure 1.

## 3. Result and Discussion

**Figure 2:**
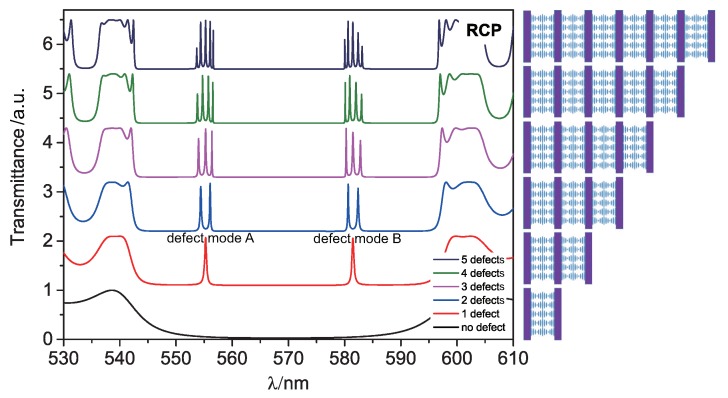
Transmission spectra (for RCP light) of composite structures with varying number of structural units. The thicknesses of the CLC and polymer layer are 4 μm and 2 μm, respectively.

Right circularly polarized (RCP) light and left circularly polarized (LCP) light are the eigenmodes for a CLC system. RCP light with wavelengths ranging between $n_{o}p$ and $n_{e}p$ will be total reflected by the CLC due to the right handedness of its helical structure, while LCP light will be transmitted. In our simulation, the incident light propagates along the helix axis of the CLCs and is indicated in Figure 1 by an orange arrow. The thickness $d_{C}$ of the CLC cells is set to approximately (since the pitch is fixed for calculation puposes) 4 μm and the thickness $d_{I}$ of the isotropic layer to 2 μm. As soon as at least one polymer layer is present, defect modes arise in the PBG as illustrated by the transmission spectrum in Figure 2, because polymer layers disturbs the periodicity of the initial CLC system.

If the CLC structure involves only one isotropic defect layer, we happen to find just two defect modes in the PBG for our model parameters. When there is more than one defect layer, splitting of those defect modes occurs. The number of splitting components is equal to the number of polymeric defect layers, analogous to the results of the TB approximation in solid state physics: Every defect layer can be seen as an “atom” with localized defect modes if the layers are well separated. If they come closer to each other, the degeneracy of the defect mode will be lifted which causes mode splitting.

This splitting phenomenon is similar to the one in three-dimensional photonic crystals [26] and in CLCs with multiple chiral defects [33]. In most previous studies, a single defect in a photonic crystal usually occupied only one or few sites. Here, the thickness of the isotropic layer is about 11 half pitches, which is equivalent to nearly 11 lattice sites in a 1D photonic crystal. This is also the reason why two defect modes appear in the PBG of our MDL-CLC structures. In the MDL-CLC structure, we assume that $E_{\Omega }\left(x\right)$ is optical field of the eigenmode associated with an individual defect site, where Ω is the eigenmode frequency. The dispersion relation for an eigenmode $E_{k}\left(x\right)$ in the MDL-CLC system with an infinite number of periods can be obtained by considering only nearest neighbor mode coupling. This results in [25]

(1) $$\omega_{k}=\Omega \left[1-\frac{\Delta \alpha }{2}+\delta \;\cos\left(kd\right)\right],$$

where the coupling factor $\delta =\beta_{1}-\alpha_{1}$ represents the coupling strength between two defect modes. The quantities $\alpha_{1}$, $\beta_{1}$ and Δα are TB parameters defined by the overlap integrals $\alpha_{1}=\int dx\;\epsilon \left(x\right)E_{\Omega }\left(x\right)\cdot E_{\Omega }\left(x-d\right)$, $\beta_{1}=\int dx\;\epsilon_{0}\left(x-d\right)E_{\Omega }\left(x\right)\cdot E_{\Omega }\left(x-d\right)$, and $\Delta \alpha =\int dx\;\left[\epsilon \left(x\right)-\epsilon_{0}\left(x\right)\right]E_{\Omega }\left(x\right)\cdot E_{\Omega }\left(x\right)$. The subscript k stands for the wavevector *k* of the eigenmodes and the dot · denotes the scalar product. In these relations, ϵ(x) is the dielectric constant of the MDL-CLC, and $\epsilon_{0}\left(x\right)$ is the dielectric constant of an individual defect structure. The value of Δα is determined by the individual defect mode. For calculation of $\alpha_{1}$, $\beta_{1}$, and δ, we need at first to analyze MDL-CLCs with a small number of periods. If the MDL-CLC structure has two defect layers, the individual defect mode Ω will split into two modes $\omega_{1}^{2}=\Omega^{2}\left(1+\beta_{1}\right)/\left(1+\Delta \alpha +\alpha_{1}\right)$ and $\omega_{2}^{2}=\Omega^{2}\left(1-\beta_{1}\right)/\left(1+\Delta \alpha -\alpha_{1}\right)$ [26]. In case of a structure with three defect layers, there exist three eigenfrequencies: $\Gamma_{1}^{2}=\Omega^{2}\left(1+\sqrt{2}\beta_{1}\right)/\left(1+\Delta \alpha +\sqrt{2}\alpha_{1}\right)$, $\Gamma_{2}^{2}=\Omega^{2}\left(1+\Delta \alpha \right)$ and $\Gamma_{3}^{2}=\Omega^{2}\left(1-\sqrt{2}\beta_{1}\right)/\left(1+\Delta \alpha -\sqrt{2}\alpha_{1}\right)$. Values of the parameters $\alpha_{1}$ and $\beta_{1}$ can be obtained from a numerical solution for the CLC structure with two defect layers, in which the small parameter Δα can be neglected. Based on the transmission spectrum shown in Figure 2, the resonant wavelength of the defect mode B is $\lambda_{11}=581.47$ nm (corresponding to Ω) for an individual defect layer. The result for a CLC with two defect layers is $\lambda_{21}=580.62$ nm, $\lambda_{22}=582.40$ nm (corresponding to $\omega_{1}$, $\omega_{2}$ respectively). Thus, $\alpha_{1}=-0.0522$ and $\beta_{1}=-0.0553$ corresponding to the defect mode B. The coupling factor is δ=−0.0031. With these values, one can then predict the wavelengths of the defect modes in CLCs with three or more defect layers. As shown in Table 1, the results of the FDTD simulation agree very well with the results of the TB approximation.

**Figure 3:**
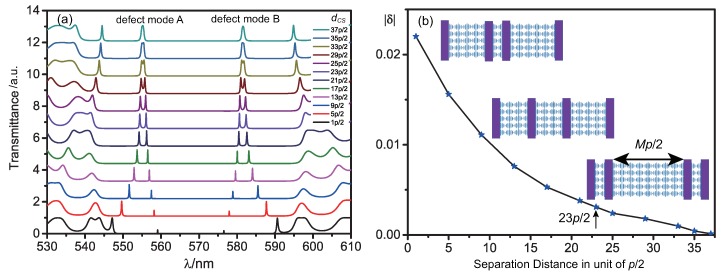
(**a**) evolution of the transmission spectra of a MDL-CLC structure with two defect layers with increasing distance between the defect layers; (**b**) relation between coupling factor |δ| and separation distance between defect layers. The whole thickness of the CLCs inside two boundary layers is 69*p/2*.

We find that when the separation distance between the two internal defect layers in the structure shown in Figure 3 decreases, the splitting of the transmission peaks in the transmission spectra become more obvious in Figure 3a. We assume the separation distance is $d_{CS}=Mp/2$, where *M* is the integer number. Here, we vary only the separation distance $d_{CS}$ of two defect layers symmetrically, while keeping the whole thickness of the CLCs inside two boundary layers to be 69*p/2*. The coupling factor δ corresponding to every case in Figure 3a can be calculated. As shown in Figure 3b, the value of |δ| decreases as the separation distance increases. Thus, the coupling strength is becoming weaker with the increasing distance between the two inner defect layers. If the separation distance is more than 37*p/2*, the coupling will be weak enough to be negligible. Besides, as illustrated in Figure 2, the separation of 23*p/2* is supposed to be suitable to obtain a reasonable coupling of defect modes in the MDL-CLC assembly.

**Figure 4:**
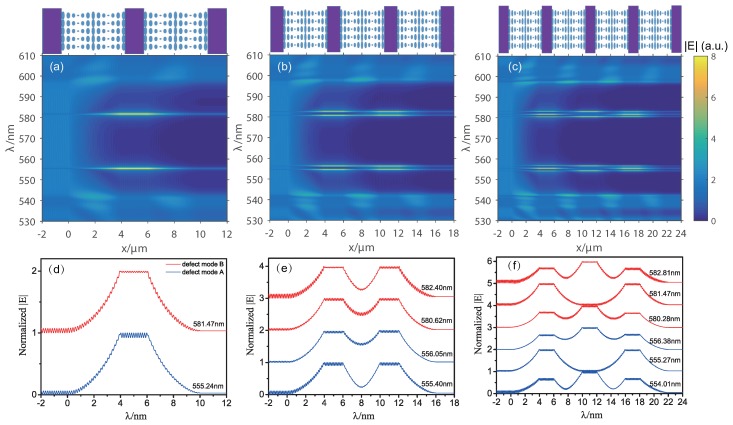
Electric field distributions for RCP light in composite structures with (**a**) one, (**b**) two, and (**c**) three isotropic layers; (**d**–**f**) electric field profiles of the defect modes at their resonant wavelengths, corresponding to (**a**–**c**), respectively.

Figure 4 shows the spatial distributions of the electric fields along the composite structures for the RCP light corresponding to Figure 2. When the structure has only one defect layer, the field of the defect mode is predominantly localized in the isotropic defect layer, as shown in Figure 4a. When the period number of the composite structure increases, the defect modes split but still remain localized mainly in the isotropic defect layers, as shown in Figure 4b,c. Detailed electric field profiles of defect modes at resonant wavelengths corresponding to Figure 4a–c are shown in Figure 4d–f.

**Figure 5:**
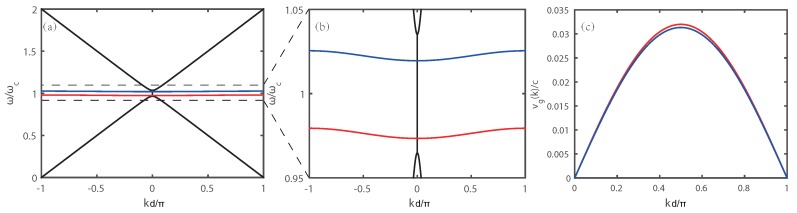
(**a**,**b**) dispersion diagrams of the CLC (black curve) and of the mini-bands formed in the MDL-CLC (blue and red curves) for RCP light in the first Brillouin zone; the quantity $\omega_{c}$ is the center frequency of the PBG; (**c**) group velocity corresponding to the mini-bands.

The spectral distribution of the defect modes broadens with increasing the number of MDL-CLC periods. If an MDL-CLC structure has an infinite number of periods, a mini-band will form in the PBG of the originally nonperturbed CLC medium. The dispersion relation of a nonperturbed CLC material is illustrated by black curves in Figure 5a,b [34]. Based on the dispersion relation described by Equation (1), one can get the dispersion diagram of the mini-band (blue and red curves) as shown in Figure 5a,b. The mini-bands originate from the defect modes A (blue curve) and B (red curve) that are located within the PBG of the CLC. The spectral width of the mini-bands is about 4 nm. The corresponding group velocity $v_{g}=d\omega_{k}/dk$ of the mini-band can be derived from Equation (1) as $v_{g}=-\Omega \delta d\sin\left(kd\right)$. It is plotted in Figure 5c.

**Figure 6:**
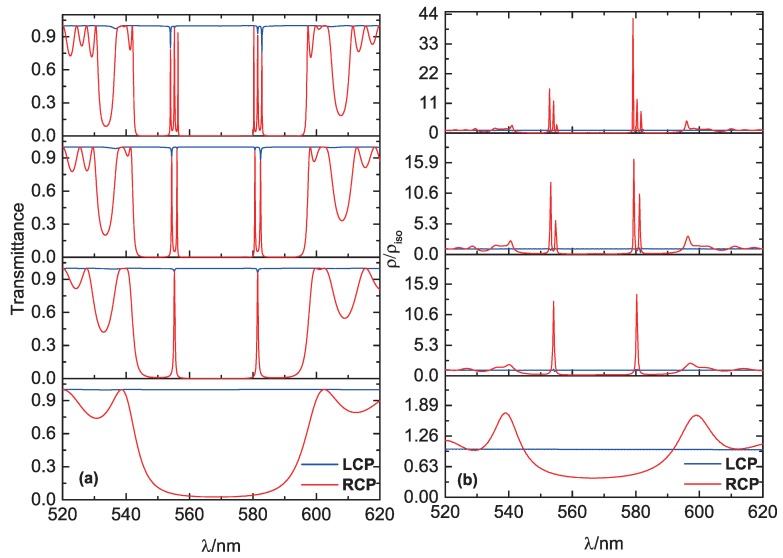
(**a**) calculated transmission spectra for RCP light (red) and LCP light (blue) for composite structures with 0, 1, 2, and 3 defect layers; (**b**) calculated relative DOS $\rho /\rho_{iso}$ for RCP and LCP light.

The Q-factor is Q=λ/Δλ, where λ is the central wavelength of the defect mode and Δλ is the full width at half maximum of the defect mode peak. If there is only one defect layer (second scheme from the bottom up in Figure 2), calculation gives Q≈1200 for defect mode B. The average Q-factor becomes larger with increasing number of periods of the MDL-CLC structure. The DOS is approximately proportional to the Q factor [35]. In a 1D photonic crystal, the DOS ρ is given by the inverse of group velocity [36]

(2) $$\rho =\frac{dk}{d\omega }=\frac{1}{D}\frac{\frac{dv}{d\omega }u-\frac{du}{d\omega }v}{u^{2}+v^{2}},$$

where *u* and *v* are the real and imaginary parts of the complex transmission coefficient t(ω) of the 1D structure, respectively. The DOS in an isotropic medium is $\rho_{iso}=n/c$, where n is the reflective index and *c* is the speed of light in vacuum. As can be seen in Figure 6, the DOS at the defect mode (for RCP light) is larger than the one at the edge of the PBG. Therefore, RCP defect mode lasing might be realized if a laser dye is doped into the CLC composite structure. The DOS peak of every defect mode corresponds to a transmission peak, while the DOS peak intensity depends not on the peak transmission intensity but the profile of the transmission peak. The DOSs at some defect modes of the MDL-CLC structures become higher with increasing number of periods as shown in Figure 6b. This can be exploited to reduce the lasing threshold. However, if the composite structure has the same thickness of the whole CLC material, the average Q-factor in a sample with multiple defects is lower than the one for a sample with a single defect. Thus, it seems in this case that a sample with a single defect would be better for low threshold lasing than one with multiple defects.

When considering experimental realization of lasing in the above-described composite structures, also scattering and absorption losses have to be taken into account [37]. If these are low enough, there might be a realistic chance to observe the splitting of the lasing modes and multi-defect lasing due to the coupling of defect modes if MDL-CLC structure contains several periodic units. As can be seen in Figure 6a, there also appear small dips in the transmission spectra for the LCP light, which are positioned at the same wavelengths as the transmission peaks for the RCP light. This interesting polarization transformation effect is also generated by the defect layers, although the initial CLC structure has no PGB for the LCP light.

## 4. Conclusions

This work investigates the coupling of defect modes in MDL-CLC composite structures. Splitting of the defect modes is observed in transmission spectra if more than one defect layer is inserted into the structure. The number of splitting components is equal to the number of the defect layers. This phenomenon is analogous to the effect of interaction in atomic systems and can be described by the TB approximation known from solid state physics. The coupling effect is stronger when the distance between defect layers becomes smaller.

The CLC is known to be very sensitive to external factors, e.g., temperature or electric field. Therefore, mode coupling in the MDL-CLC assemblies can be readily controlled by various external stimuli. Hence, it seems to be interesting to explore MDL-CLC structures in particular with two applications in mind: multiwavelength filters in photonic devices and multimode lasing. The latter can be realized if an optically active material (e.g., a laser dye) is added to the structure and because the splitting of the optical emission modes can be tuned according to specification. 

## Figures and Tables

**Table 1:** Comparison of resonant wavelengths of optical defect modes of the CLC structure with three defect layers calculated by the FDTD-based numerical simulation and calculated analytically on the basis of the TB model.

	FDTD Simulation Result/nm	TB Method/nm
$\lambda_{31}$	580.28	580.29
$\lambda_{32}$	581.47	581.47
$\lambda_{33}$	582.81	582.81

## Abbreviations

The following abbreviations are used in this manuscript:

CLC	Cholesteric liquid crystal
MDL-CLC	a system of multiple defect layers in a Cholesteric liquid crystal
TB	Tight binding
DOS	Density of states
FDTD	Finite difference time domain
RCP	Right circularly polarized
LCP	Left circularly polarized
